# Chikungunya Infection in Travelers

**DOI:** 10.3201/eid1210.060495

**Published:** 2006-10

**Authors:** Patrick Hochedez, Stephane Jaureguiberry, Monique Debruyne, Philippe Bossi, Pierre Hausfater, Gilles Brucker, Francois Bricaire, Eric Caumes

**Affiliations:** * Hôpital Pitié-Salpêtrière, Paris, France;; † Laboratoire Pasteur Cerba, Cergy Pontoise, France

**Keywords:** Chikungunya virus, Exanthema, Rash, Travel, Indian Ocean Islands, Reunion, Arthralgia, Dispatch

## Abstract

The largest described outbreak of chikungunya virus has been occurring on the islands of the southwest Indian Ocean since March 2005. We describe the manifestations of chikungunya virus infection in travelers returning from these islands, with focus on skin manifestations.

Chikungunya virus is an arthropodborne virus (genus *Alphavirus*, family *Togaviridae*) (*1*). It was first isolated in Tanzania in 1953 (*2*). Since then, chikungunya outbreaks have been reported in Africa and Asia. Transmission to humans occurs through bites of *Aedes* (mainly *Aedes aegypti* and *A. albopictus*) mosquitoes (*3**–**7*).

An outbreak of chikungunya occurred in the Comoro Islands in early 2005; since then, the virus has circulated to other islands in the Indian Ocean. The first case of chikungunya infection was identified in the Indian Ocean island of Reunion in March 2005. After a period of lower transmission during winter, case numbers increased dramatically with the arrival of the Southern Hemisphere summer in December (*8*). The Reunion epidemic is the largest ever described; ≈150,000 cases were reported from March 2005 through February 2006. We describe skin manifestations of this chikungunya infection in travelers returning from the area.

## The Study

All adult travelers who came to our tropical diseases unit from March 2005 through February 2006 were prospectively included in this study if they had symptoms of chikungunya virus infection after traveling to any island in the southwest Indian Ocean (Comoro, Mauritius, Mayotte, Reunion, Seychelles). Children <5 years of age were not included because no pediatricians practiced in our hospital. Chikungunya virus was suspected if fever, joint pain, or skin eruption was present. Diagnosis of chikungunya infection was confirmed by an immunocapture ELISA derived from a yellow fever test by using a goat anti-human immunoglobulin (Ig) M antibody (Sigma, Saint Louis, MO, USA), an inactivated cell-culture–grown chikungunya virus and a mouse anti-chikungunya hyperimmune ascitic fluid (Institut Pasteur, Lyon, France), and a horseradish peroxidase–labeled antimouse IgG conjugate (Sigma) (*9*). Other blood tests were performed according to the discretion of the physician in charge of the patient.

During the study period, chikungunya infection was confirmed serologically in 22 patients (14 female, 8 male); median age was 47 years (25–72 years). Seventeen patients had returned from Reunion (77%), 3 from Comoro (14%), and 2 from Mauritius (9%). Twelve (55%) patients were tourists, 8 (36%) were islanders settled in France but returning from visiting their relatives, and 2 (21%) were French residents in these islands. The median duration of stay (residents excluded) was 21 days (2–90 days). Symptoms appeared in 19 (86%) patients while they were abroad and within 3 days of return in 3 patients (14%). The median lag time between onset of symptoms and consultation was 13.5 days (range 2 days–9 months).

Fever and joint pain were noted in all patients. The median duration of fever was 4 days (range 2–7 days), and joint pain was mainly distal and symmetric, involving wrist (18 patients, 81%), ankles (17 cases, 77%), phalanx (16 patients, 73%), knees (14 patients, 64%), and elbows and shoulders (4 patients each, 18%). The other extradermatologic signs were asthenia in 17 patients (77%), headache in 13 (59%), muscle pain in 12 (55%), swelling in 10 (45%), peripheral lymphadenopathy in 9 (41%), bleeding from the nose or gums in 4 (18%), nausea or vomiting in 3 (14%), and eyesight trouble in 1 (4%). In the 6 patients seen during the first week of symptoms (acutely ill patients), lymphopenia was present in 67%, thrombocytopenia in 50%, and increase of alanine aminotransferase/aspartate aminotransferase in 67%.

Skin manifestations occurred in 17 patients (77%). Because skin eruption occurs during the first week of the disease, however, only 6 (27%) patients with these signs were seen at the time of consultation. All 6 had a generalized exanthema consisting of noncoalescent macular or papular lesions (Figure). The elementary cutaneous element was an erythematous macule in 5 patients and maculopapules in 1. The number of macules was >50, and the anatomic location of the rash was abdomen, thorax, back, and limbs. No lesions were seen on the face; palms and soles were involved in 3 patients. Islands of normal skin were seen in 5 patients. An aphthoid lesion was seen in 1 patient. Pruritus of the skin or a burning sensation was reported in 3 patients. Bleeding from the nose or gum, purpura, petechiae, ecchymosis, and bullous lesions were not observed. Erythema followed the onset of fever by 1 to 2 days, lasted 3–7 days, and disappeared without scaling in all cases, regardless of the patient's skin color.

**Figure:**
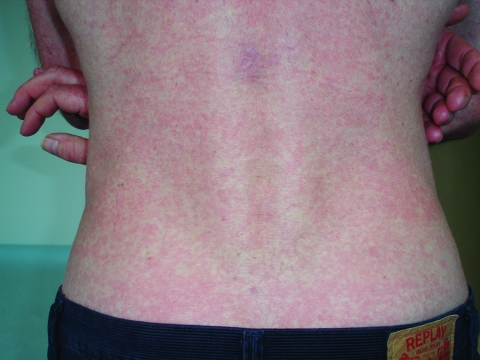
Erythematous maculopapuar exanthema with islands of normal skin on the back.

The main complaint after the first week was persistent arthralgia. The treatment was exclusively symptomatic: pain killers and nonsteroidal antiinflammatory medications.

The dramatic ongoing epidemic of chikungunya infection in the southwestern part of the Indian Ocean, particularly Reunion Island, provided the opportunity to describe the skin manifestations of this infection among travelers. The main characteristic of the skin eruption was a generalized macular erythematous exanthema, often with islands of normal skin and a sensation of pruritus. The infection was also characterized by a short incubation period (1–12 days). Most patients had fever, arthralgia, exanthema, asthenia, myalgia, and headache.

## Conclusions

Among travelers returning from the tropics, febrile exanthema associated with arthralgia is not specific to chikungunya and may be observed with several other viral and bacterial infections and adverse cutaneous reactions (*10*). Chikungunya and dengue infections are probably the most difficult to differentiate. The viruses are transmitted by the same mosquito species; disease-endemic areas are nearly the same in Asia, Africa, and the Indian Ocean; and clinical symptoms are similar. In addition, simultaneous coinfection with chikungunya and dengue viruses has been reported (*5**,**11*). In the only published study comparing chikungunya and dengue clinical manifestations in Thailand, the onset of symptoms was more abrupt; the febrile course was shorter; and maculopapular rashes, conjunctival injection, and arthralgia were significantly more frequent than in dengue (*12*). Shock and gastrointestinal hemorrhages occurred only in dengue patients, but the tourniquet test did not help to differentiate chikungunya from dengue (*12*). The skin manifestations reported in our study are similar to those described for classic dengue fever infection, which is characterized by a pruritic, macular or a maculopapular rash in which small islands of normal skin are spared (*12*).

The *Alphavirus* genus consists of 30 species of arthropodborne viruses (*1**,**13*). Of these, 6 mosquitoborne viruses are associated with fever, arthralgia, and rash: the Ross River and Barmah Forest viruses in the South Pacific, o'nyong-nyong and Sindbis viruses in tropical Africa, chikungunya virus in Africa and Asia, and Mayaro virus in South America. Symptoms are usually of short duration, ≈1 week, and complete recovery is the rule, except in some cases of chikungunya infection, in which arthralgia may last for months (*14**,**15*). Indeed, 7 patients in our study had at least a 1-month duration of arthralgia; 1 patient consulted us 9 months after the onset of disease. The clinical manifestations reported in this study illustrate the difficulty in differentiating chikungunya infection from classical dengue fever but may help to differentiate this infection from other causes of febrile exanthema in travelers.
